# Vascular Precision Medicine: A Ray of Sunshine for the Therapies of the Present and Future

**DOI:** 10.1177/00033197251341773

**Published:** 2025-05-31

**Authors:** Gaetano Lanza, Carlo Setacci

**Affiliations:** 1 Department of Vascular Surgery, IRCCS Policlinico Multimedica, Sesto San Giovanni, Milan, Italy; 2 Department and Residency Program in Vascular Surgery, University of Siena, Siena, Italy

**Keywords:** vascular diseases, precision medicine, big data, artificial intelligence

Medical sciences in the last decades of the last century and in the first years of the current century has witnessed an enormous development of knowledge.

Above all Surgery, with the evolution of mini-invasiveness (laparoscopic, robotics, endovascular approach) and Radiology (with the enormous development of diagnostic technologies) are experiencing an evolution that is as positive as it is, perhaps, unexpected.

We have therefore witnessed an evolution of scientific thought which has moved from personal or small group experiences to a more rational approach to research, supported by incontrovertible elements which certified the validity of some theses which were initially the expression of completely personal beliefs. We therefore arrive at evidence-based medicine which goes beyond the now outdated concept of experience-based medicine.

Further development of knowledge was brought about by the birth of Precision Medicine^1^(PM). No longer a generic approach to the identified pathology (the “so called” protocols applicable to all carriers of a certain disease) but a solution customized to the characteristics of the patient.

The same pathology can have a completely different impact on the prognosis of the single patient. The body’s reaction is individual and cannot be subjected to generic protocols valid for everyone.

PM is an emerging practice of medicine that uses an individual genetic profile to guide decision made in regard to the prevention, diagnosis and treatment of disease.

Inevitably, when we talk about PM we cannot overlook the fundamental role played by Artificial Intelligence (AI) and the correct analysis of Big Data (BD).^2–6^ An area of interest of AI is the ability to predict the risks and complications associated with Vascular Surgeries. By analyzing clinical data such as patient history, laboratory test results and diagnostic images, machine learning algorithms can identify patients at risk of developing postoperative complications. This allows healthcare providers to take preventive pleasures and personalize treatments to maximize clinical outcomes.

Imaging diagnostic represent a critical phase in the treatment offered to patients in Vascular Surgery, helping to confirm the diagnosis, evaluate the prognosis, and plan the surgical intervention. AI approaches can assist in optimizing image segmentation and pattern identification, as well as automating repetitive tasks, increasing repeatability and reducing computation time. Various AI-derived algorithms, for example, have been used to improve aortic aneurysm segmentation, allowing for a detailed assessment of the aneurysm’s geometry and morphology.^7^ Machine learning has also been used to create fully automated pipelines for the detection and measurement of vascular calcifications in computed tomography images.^8^ AI has promising applications in image segmentation, automation, data analysis from medical records, facilitating and improving data collection and quantitative measures in large patient datasets. The risk to patients and the outcomes of the operation can be better assessed using a combination of these techniques. For example several machine learning algorithms have been designed to assess the risk of aortic aneurysm development and rupture or to predict outcomes after surgical aneurysm repair.^8^ A recent study^9^ revealed significant cultural variations in the treatment of juxtarenal AAAs, with Vascular Surgeons recommending continuous monitoring, endovascular surgery or open surgery for the same patient. This underscores the critical need for new technologies to assist surgeons in determining the best treatment strategy. AI could potentially categorize patient status, better estimate the risk of pre-and post-operative complications and advise surgeons on the most appropriate surgical method, enabling the formulation of multivariable scores that incorporate clinical, biological and imaging parameters.

Image segmentation and risk classification systems have been developed for patients with carotid artery stenosis.^10^

Lee et al.^11^ built several models to identify peripheral arterial disease (PAD) that frequently goes undiagnosed. They compared their models to standard logistic regression ones and showed that AI was able to produce more accurate predictive algorithms, thought either to identify patients with PAD and to predict mortality. As a matter of fact, PAD population is still currently missing strong risk prediction models. For example Ross et al.^12^ used a variety of patient data, including genomic, imaging and socio-economic variables not obtainable within the usual clinical research pathway to identify patients at risk.

By analyzing BD, AI is constantly learning and introduces the concept of predictive medicine, that has already been tested and appreciated in some specific areas, such as medical imaging, cancer care and dermatology. AI may allow to build diagnostic and therapeutic programs, individualized to each patient thanks to the large analysis of his and her clinical, biological and even genetic characteristics.

The management of BD may result in a useful repository of patients advanced clinical data, provided that hospitals will be able to make a true “revolution” by introducing new professions and pedagogical figures aimed to guide its use into the clinical environment. The interaction with medical practitioners is a key-point to help engineers to avoid irrelevant data collection, misleading analyses and possible confounding indications.^13,14^
